# Preeclampsia and Its Maternal and Perinatal Outcomes in Pregnant Women Managed in Bahrain’s Tertiary Care Hospital

**DOI:** 10.7759/cureus.24637

**Published:** 2022-05-01

**Authors:** Shazia Tabassum, Abeer AlSada, Noora Bahzad, Noora Sulaibeekh, Abida Qureshi, Nawal Dayoub

**Affiliations:** 1 Obstetrics and Gynaecology, Bahrain Defence Force Hospital, Royal Medical Services, Riffa, BHR; 2 Obstetrics and Gynaecology, Assisted Reproduction in Gynaecology Center, University of Bristol, Westminster, GBR

**Keywords:** pregnancy induced hypertension, pregnancy, pregnancy complication, pregnancy outcome, pre-eclampsia

## Abstract

Objective: Hypertensive disorders during pregnancy being the leading causes of maternal and fetal morbidity and mortality remains a serious health issue worldwide due to the high rate of adverse maternal outcomes and close association with neonatal morbidity and mortality. The purpose of our study was to ascertain the perinatal outcomes of preeclampsia (PE) in a tertiary care hospital in Bahrain.

Methods: A retrospective cohort study was conducted from January 2018 to December 2019 in the department of Gynecology and Obstetrics in Bahrain Defense Hospital. The process of data collection included a baseline review of all women who had delivered during the study period in order to identify those with PE. Additionally, the postdelivery records of the mothers and newborns were reviewed to identify relevant maternal and neonatal outcomes.

Results: During the research period, records revealed 142 patients with PE with a rate of 1.95%. The mean gestational age at diagnosis was 35.61 (± 3.69) weeks, ranging between 20 and 42 weeks. The mean birth weight was 2.64 ± 0.79 kg, ranging from 0.5-4.5 kg. Furthermore, most babies had an Apgar score of 9 at 5 minutes. The preterm delivery rate was (16.3%) and intrauterine growth restriction (IUGR) was seen in 19 patients (13.5%) and it was significantly higher in patients who presented between 30 and 34 gestational weeks P < 0.001. Twenty-one infants were admitted to the NICU primarily for prematurity and low birth weight.

There was only one early neonatal death of a hydrops baby. One infant was stillborn with extreme prematurity at 24 weeks+4 days. Maternal complications included five abruption placentae (3.5%) cases, five HELLP syndrome (hemolysis, elevated liver enzymes, and low platelet count) cases (3.5%), four eclampsia (2.8%) cases, and four patients had ICU admission. Other maternal complications included acute renal failure (ARF) in two patients (1.4%), pulmonary edema in one patient, and peripartum cardiomyopathy in one patient. Data showed that adverse pregnancy outcomes were significantly more common in those with PE as compared to other pregnant populations.

Conclusion: Preeclampsia causes a remarkable increase in adverse maternal and perinatal outcomes as compared to the normotensive pregnant population. A regular goal-oriented clinical audit into perinatal morbidity and mortality associated with this condition and an active multidisciplinary approach to the management of pre-eclampsia patients in the hospital might improve the clinical outcomes.

## Introduction

Preeclampsia (PE) is a progressive multisystem illness that manifests as hypertension and proteinuria after 20 weeks of gestation or postpartum, or as hypertension with substantial end-organ failure with or without proteinuria [1]. This develops in around 10%-25% of patients with pregnancy-induced hypertension. Another condition known as superimposed preeclampsia is defined as preeclampsia occurring in a woman who already has chronic hypertension. This is characterized by worsening of hypertension, the onset of proteinuria or an abrupt increase in proteinuria, and/or significant new end-organ dysfunction [1]. Pregnant women who have a history of preeclampsia or chronic hypertension are at risk of developing preeclampsia. In these patients, prophylactic administration of aspirin before 16-week gestation has been proven to reduce the risk of preeclampsia [2].

About 5%-7% of pregnant women worldwide have preeclampsia, and it is the main cause of both maternal and fetal morbidity and mortality [3]. Hypertensive disorders are the second most common cause of maternal death after hemorrhage in the developed world [4]. According to the World Health Organization (WHO), hypertensive disorders alone are responsible for 70,000 maternal fatalities and 500,000 newborn deaths annually worldwide [5]. A study by Subki et al. from Saudi Arabia showed a 2.4% prevalence of hypertensive disease during pregnancy, and PE was the most prevalent hypertensive disorder with 54.9%. The study showed that it is important to detect the condition early in the pregnancy so that pregnancy can be followed with close monitoring to prevent complications [6]. Therefore, it is crucial to develop and implement a protocol for preeclampsia to be used by obstetric units, and it should be followed by all healthcare workers involved in the care of such patients, as shown in our study site all protocols are followed by current international guidelines from screening and further management of these high-risk pregnancies with obviously improved outcomes.

To minimize maternal and neonatal morbidity, preeclampsia is managed by an evaluation of maternal and fetal status alongside the decision of timely delivery, which most guidelines advocate at 37 weeks [7]. In severe preeclampsia, expectant management can be considered until 34 weeks of gestation if strict inclusion criteria and appropriate resources are met. However, maternal and fetal well-being should be monitored closely in these patients, and delivery should be considered at any moment when maternal or fetal condition deteriorates [8]. Regardless of gestational age, signs of severe hypertension, maternal end-organ malfunction (e.g., central nervous system symptoms, hepatic abnormality, thrombocytopenia, renal abnormality, and pulmonary edema) or unsatisfactory fetal well-being tests generally necessitate prompt delivery [1].

According to data from the Hospital Corporation of America, the timely administration of antihypertensive medications reduces the risk of fatal cerebral bleeding, renal failure, and injury, as well as cardiovascular morbidity and mortality (specifically heart failure and myocardial ischemia) [9,10]. The treatment of acute hypertension may also require intravenous medicine. These facts highlight the need to establish and adhere to proper local and national protocols. Labetalol is the first-line antihypertensive medicine, which acts as alpha-and beta-adrenergic antagonists in combination. Methyldopa, labetalol, and calcium channel blockers (i.e., nifedipine) are among oral antihypertensive drugs. Magnesium sulfate has been the medicine of choice for more than 70 years to prevent eclamptic seizures because of its superior safety and efficacy. The WHO recommends magnesium sulfate for the treatment of eclampsia [11].

This paper discusses a retrospective cohort study performed from 2018 to 2019 which aimed to ascertain the total number of preeclampsia patients, their in-patient management, and their primary pregnancy outcome, which include eclampsia, HELLP syndrome (hemolysis, increased liver enzymes, low platelets), pulmonary edema, cerebral hemorrhage, renal failure, preterm deliveries, intrauterine growth restriction (IUGR), and cesarean sections.

## Materials and methods

A retrospective cohort study was carried out between January 2018 and December 2019 in the Department of Gynecology and Obstetrics at Bahrain Defense Force Hospital. The study population included all pregnant women who presented with new-onset hypertension with proteinuria or headache and/or epigastric discomfort at 20 weeks of gestation or later. Hypertension was defined as having two blood pressure (BP) measurements taken 4 hours apart with a systolic BP ≥ 140 mmHg or diastolic BP ≥ 90 mmHg. Proteinuria was defined as the presence of 1+ or more proteins on the reagent strip and confirmed in the laboratory. The features of severe preeclampsia included systolic BP ≥ 160 mmHg or diastolic BP ≥ 110 mmHg, urine protein ≥ 3+, persistent headache, blurred vision, persistent epigastric discomfort, high creatinine, and disordered liver enzymes. Eclampsia was characterized by preeclampsia-like signs and symptoms and grand mal seizures that are unrelated to other brain illnesses.

Electronic health records of all patients admitted with preeclampsia were reviewed for antenatal care (ANC), intrapartum care, and immediate postnatal care (PNC), and their pregnancy outcomes were recorded and analyzed. The sample size was determined based on the prevalence of preeclampsia at the study site. This study included all pregnant women with preeclampsia, regardless of ethnicity, parity, and singleton/multiple pregnancies. It also included patients with superimposed preeclampsia. Patients who developed preeclampsia before 34 weeks were labeled as early-onset preeclampsia. The key outcome variables were unfavorable perinatal and maternal outcomes that occurred during the study period or within seven days of the participant’s hospitalization or delivery. Preterm and low birth weight babies, IUGR, antepartum stillbirths, intrapartum stillbirths (i.e., fetal death before the onset of labor), and admission to the neonatal intensive care unit (NICU) or neonatal mortality within seven days of discharge (whichever occurred first) were all included in the definition of an adverse pregnancy outcome. The mother’s adverse outcomes included admission to an intensive care unit (ICU), HELLP syndrome, pulmonary edema, acute renal failure (ARF), eclampsia, peripartum cardiomyopathy, and primary postpartum hemorrhage (i.e., estimated blood loss of >500 mL after vaginal delivery or >1000 mL after cesarean delivery within 24 hours after delivery).

Statistical analysis

Continuous variables were expressed as the continuous mean and standard deviation, whereas discrete variables were expressed as frequency and percentages. Descriptive analysis was done. Associations between discrete variables were calculated using the Chi-Square test or Fisher’s exact test, as appropriate. Statistical significance was set at p < 0.05. Data analysis was performed by using statistical analysis for social science (SPSS) version 23.00 (IBM Corp., Armonk, NY) and Microsoft Excel.

Ethical consideration 

Ethical approval was obtained from the ethical committee of the Bahrain Defence Force (BDF) Hospital of the Royal Medical Services. Consent from the patients has been waived off from the ethical committee of the institute. The ethical committee of the BDF hospital issued approval BDF/R&REC/2019-479. 

## Results

During the research period, there were 7,286 deliveries, and 142 patients presented with preeclampsia with a rate of 1.95%. More than half of the patients (61%) were Bahraini, while the rest were from other countries. The mean maternal age of patients with preeclampsia was 32.27 (± 6.42) years, ranging between 19 and 46 years. There were 121 patients (85.8%) booked for ANC. Unexpectedly, only 30 patients (21.3%) were nulliparous, and 32 (22.7%), 26 (18.4%), 21 (14.9%), and 8 (5.7%) were para 1, 2, 3, and 4, respectively, whereas the remaining were para 5 and more (multiparous). Around (26.2%) of patients had a history of preeclampsia, 24.1% had pre-pregnancy hypertension, and 15% had merely a prior history of pregnancy-induced hypertension (PIH). Diabetes mellitus was present in 15 patients (10.6%). The diagnosis was made mostly in the antenatal period with 14% of patients diagnosed intrapartum and 2.1% diagnosed postpartum. The mean gestational age at diagnosis was 35.61 (± 3.69) weeks, ranging between 20-42 weeks (Table 1).

**Table 1:** Demographic characteristics of patients (n = 141) PIH: Pregnancy-induced hypertension; DM: Diabetes mellitus

	Patients with preeclampsia (n = 141)
Maternal age, years mean ± SD	32.27 ± 6.42
Range, years	19–46
Nationality:	
Bahraini	86
Pakistani	14
Syrian	17
Indian	8
Others	16
Parity:	
0	30
1	32
2	26
3	21
4	8
5	10
6	7
7	2
8	4
10	1
Previous medical history of:	
PIH	21
Preeclampsia	37
Pre-pregnancy hypertension	34
DM	15
Time of diagnosis:	
Intrapartum	21
Postpartum	3
Antenatal	117
Gestational age, weeks mean ± SD	35.61 ± 3.69
Range of gestational age, weeks	20–42
Booking status:	
Booked	121
Unbooked	20

One-third of patients presented with headache (29.1%), whereas epigastric pain and blurred vision were the initial symptoms in 17.7% and 13.5%, respectively. Moreover, 2.8% of patients had a pre-admission eclamptic fit. Patients had a mean body mass index (BMI) of 33.19 ± 7.27 kg/m^2^, which was similar across patients who presented at different gestational ages (p = 0.68; Table 2). More than half of patients (61%) presented with diastolic BP of 90-100 mmHg, one-third presented with a diastolic BP of >100-120 mmHg, and only 2.1% presented with diastolic BP of >120 mmHg. One-third of patients had proteinuria of 1+ upon initial assessment, whereas only 1.4% had proteinuria of more than 4+. The mean hemoglobin level was 11.6 ± 1.25. Abnormal liver function tests were noted in 20.6% of patients, but only 3.5% had low platelet levels (<100).

**Table 2:** Signs and symptoms and laboratory parameters BMI: Body mass index; HB: Hemoglobin; LFT: Liver function test

	Patients with preeclampsia (n = 141)	%
Symptoms		
Headache	41	29.1
Blurred vision	19	13.5
Epigastric pain	25	17.7
Eclamptic fit pre-admission	4	2.8
BMI kg/m^2 ^mean ± SD	33.19 ± 7.27	
Range	17–61	
Blood pressure (BP)		
Systolic BP >200mmhg	3	2.1
Diastolic BP 90–100 mmHg	86	61
Diastolic BP >100–120 mmHg	42	29.8
Diastolic BP >120mmhg	3	2.1
Blood results		
HB gm/dl mean ± SD	11.60 ± 1.25	
Range	8.5–17	
Low platelets <100	5	3.5
Abnormal LFTs	29	20.6
Raised creatinine	6	4.3
Uric acid umol/L mean ± SD	356.55 ± 92.93	
Range	221–750	
Urine protein		
1+	43	30.5
2+	41	29.1
3+	11	7.8
4+	2	1.4

During their admission, 71 patients received magnesium sulfate, which was mainly used as prophylaxis in 68 patients (48.2%) and for therapeutic purposes in three patients (2.1%). The main choice for antihypertensive drugs was labetalol (76 patients, 53.9%) followed by methyldopa (53 patients, 37.6%). Other options such as hydralazine, calcium channel blockers, and nitroglycerine were used in around 10% of patients as detailed in Table 3. The mean gestational age at delivery was 36.48 ± 3.36 weeks. One-fourth of patients had to be induced to start labor, whereas more than half (58.2%) delivered via cesarean section with a mean hospital stay of 5.17 ± 2.6 days.

**Table 3:** Management at admission and MOD IOL: Induction of labor; MOD: Mode of delivery; SVD: Spontaneous vaginal delivery

	Patients with preeclampsia (n = 141)	%
Magnesium sulfate		
Prophylactic	68	48.2
Therapeutic	3	2.1
Antihypertensive drug:		
Labetalol	76	53.9
Methyldopa	53	37.6
Hydralazine	9	6.4
Calcium channel blockers	5	3.5
Nitroglycerine	2	1.4
Gestational age at delivery	36.48 ± 3.36	
Range	24–42	
IOL	36	25.5
MOD		
SVD	59	41.8
Assisted vaginal delivery	2	1.4
Emergency cesarean	46	32.6
Elective cesarean	36	25.5
Hospital stay, days	5.17 ± 2.62	
Range, days	1–14	

The mean birth weight was 2.64 ± 0.79 kg, ranging from 0.5-4.5 kg with a nearly equal prevalence of fetal sex. Furthermore, most babies had an Apgar score of 9 at 5 minutes. the preterm delivery rate was (16.3%) and IUGR was seen in 19 patients (13.5%) and was significantly higher in patients who presented between 30 and 34 gestational weeks P < 0.001. NICU admission was in 21 babies and the main cause for admission was prematurity and low birth weight. There was one early neonatal death of a hydrops baby. One baby was stillborn with extreme prematurity at 24 weeks+4 days. Maternal complications included five abruption placenta (3.5%), five HELLP syndrome cases (3.5%), fourth eclampsia (2.8%), and four patients had ICU admission. Other complications included ARF in 2 patients (1.4%), pulmonary edema in one patient, and peripartum cardiomyopathy in one patient (Table 4).

**Table 4:** Maternal and fetal outcomes IUGR: Intrauterine growth restriction; HELLP: hemolysis, increased liver enzymes, low platelets; ICU: Intensive care unit

	Patients with preeclampsia (n = 141)	%
Fetal sex		
Male	70	49.6
Female	71	50.4
Birth weight	2.64 ± 0.79	
Range	0.5–4.5	
Hydrops fetalis	1	0.7
Stillbirth	1	0.7
IUGR	19	13.5
Apgar score		
<5 at 1 minute	6	
<5 at 5 minutes	2	
Maternal complications		
Iatrogenic preterm delivery	23	16.3
Abruptio placenta	5	3.5
HELLP syndrome	5	3.5
Acute renal failure	2	1.4
Eclampsia	4	2.8
Peripartum cardiomyopathy	1	0.7
Pulmonary edema	1	0.7
Postpartum hemorrhage	2	1.4
ICU admission	4	2.8

The preterm delivery rate was significantly higher when patients presented at a gestational age of 25-29 weeks compared to 30-34 weeks (66.7% vs. 50%, p < 0.001; Table 5).

**Table 5:** Patient characteristics according to gestational age at diagnosis IUGR: Intrauterine growth restriction; BMI: Body mass index

	Gestational age at diagnosis of preeclampsia (weeks)					P-value
	20–24	25–29	30–34	35–39	>39	
BMI						0.68
≤30	1 (50)	3 (50)	11 (34.4)	26 (30.2)	6 (40)	
>30	1(50)	3 (50)	21 (65.6)	60 (69.8)	9 (60)	
IUGR	0 (0)	1 (16.7)	13 (40.6)	3 (3.5)	2 (10.5)	<0.001
Preterm delivery	1 (50)	4 (66.7)	16 (50)	2 (2.3)	0 (0)	<0.001

## Discussion

Hypertensive disorders in pregnancy lead to poor maternal and fetal outcomes compared to pregnancy outcomes in healthy women. The early diagnosis and timely management of this condition can improve outcomes and reduce the risk of complications. The exact pathophysiology behind this condition is still being studied, but it is generally accepted that disturbed placental development in certain pregnancies leads to cellular, molecular, immunological, and vascular changes [12]. This process leads to placental hypoperfusion, which leads to the release of antiangiogenic factors into the maternal bloodstream and impairment of maternal systemic endothelial function, ultimately resulting in preeclampsia [13].

In this retrospective cohort study, 7286 patients were initially included, with 141 diagnosed with preeclampsia, or around 1.95%. This percentage is less than the global prevalence of preeclampsia, which is estimated to be 4.6% (95% {CI}: 2.7%-8.2%) [14]. In our study, preeclampsia was more common in middle-aged women, with the mean age being 32.27 years, which is comparable to the average age of 31.3 ± 6.7 years in Saudi Arabia. However, our study found preeclampsia to be less common in multigravida patients compared to the study in Saudi Arabia [6].

The history of preeclampsia, chronic hypertension, gestational diabetes, antiphospholipid syndrome, and morbid obesity (BMI > 35 kg/m^2^) are all significant risk factors for preeclampsia. Additionally, primigravida, advanced maternal age, multiple pregnancies, chronic renal disease, and a family history of preeclampsia all increase the risk of developing preeclampsia [15]. The most significant risk factors for preeclampsia identified in our study were a previous history of PIH, a history of preeclampsia, diabetes mellitus, and obesity [16].

Keeping BP at an optimal level is important in preeclampsia. While managing mild to moderate hypertension, physicians should consider the potential risk of developing severe hypertension, the potential benefit of preventing severe hypertension, and the patient’s comorbidities and symptoms [17]. The practice bulletin of the American College of Obstetricians and Gynecologists recommends the use of methyldopa and labetalol as the first-line treatment for hypertensive disorders of pregnancy (HDP) [18]. Similarly, the main antihypertensives of choice in our patients were also labetalol and methyldopa, used in 53.9% and 36.6% of patients, respectively.

Magnesium sulfate remained the drug of choice in patients at the study site for prevention and treatment of eclampsia, with 68 (48.2%) and 3 (2.1%) patients receiving this for prophylaxis and for its therapeutic effect after the onset of eclampsia, respectively. According to the Magpie (Magnesium Sulfate for the Prevention of Eclampsia) trial, women who received magnesium sulfate had a considerably decreased risk of developing eclampsia [19]. According to Cochrane reviews, magnesium sulfate is preferable over diazepam and phenytoin for the treatment of eclampsia [20]. Overall in this study, the maternal complication rate was 14.18%. The rate of cesarean delivery was also high at 51.06%. Fortunately, there was no case of maternal mortality in this study through a retrospective study conducted at four hospitals in Haiti over a period of three years (2012 to 2014) reported a five times higher likelihood of maternal death due to preeclampsia [21]. To minimize the morbidity and potential mortality associated with preeclampsia, evidence-based therapy, and excellent hospital care are important. The cost incurred because of the care of such cases, especially due to the length of hospital stay and ICU admission, can be difficult to minimize. The average hospital stay of patients in our study was 1 to 14 (5.17 ± 2.62) days, which can place a burden on resources.

In a study of fetal outcomes in hypertensive disorder in pregnancy, Bridwell et al. found babies that were small for gestational age were four times more common among inpatients with PE [22]. In our study period, the average birth weight of the babies of PE patients was 2.64 kg, with a significant association between IUGR and PE diagnosed between 30 and 34 weeks of gestation. When a patient presents with PE after 34 weeks of gestation, the effect on fetal growth is negligible. Surprisingly, in this analysis, IUGR was diagnosed in only one out of nine cases who were admitted at less than 30 weeks of gestation. This reflects the severity of early presentation, as most cases would have to undergo early intervention and delivery. The preterm delivery rate was significantly higher in patients who presented between 25 and 29 weeks of gestation, with a rate of 66.7%.

Although there was a significant number of obese patients in our study, there were no adverse outcomes seen with obesity. BMI at presentation had no statistically significant correlation with gestational age (p > 0.05). This finding needs to be interpreted with caution, as this could be due to the study’s small sample size. It is essential to highlight that the outcomes of patients with preeclampsia are not always satisfactory. Between January and February 2013, a cross-sectional study conducted at Korle Bu Teaching Hospital in Accra, Ghana, demonstrated a considerable rise in preeclampsia patients’ perinatal morbidity and mortality [23]. Meanwhile, our study was conducted at a tertiary referral center with free availability of surfactants and high-quality NICU care. Thus, it is possible that our results do not accurately represent the Bahraini population. The limitations of our study are a short study period, a small sample size, and a single-center study. To better understand the dynamics of maternal and perinatal outcomes linked with PE, larger longitudinal studies with a longer duration should be conducted. Further studies are needed to analyze the long-term consequences on maternal and neonatal health, explore the pathology of preeclampsia, and study the effects of early screening and preventive strategies.

## Conclusions

This study establishes the baseline for maternal and perinatal outcomes associated with PE in Bahrain, which can be used to guide future research. The findings of this study provide a general overview of the maternal and perinatal health concerns associated with PE and the influence of satisfactory care in a tertiary hospital on better results for this condition. Our key findings determined a significant burden of perinatal morbidity and mortality associated with PE in the obstetric population. We recommend a regular goal-oriented clinical audit into perinatal morbidity and mortality associated with PE and input of a multidisciplinary approach to the management of these disorders in the hospital to improve the clinical outcomes.
